# HOXA5 inhibits the proliferation and neoplasia of cervical cancer cells via downregulating the activity of the Wnt/β-catenin pathway and transactivating TP53

**DOI:** 10.1038/s41419-020-2629-3

**Published:** 2020-06-04

**Authors:** Hong-Mei Ma, Nan Cui, Peng-Sheng Zheng

**Affiliations:** 1 Department of Reproductive Medicine, The First Affiliated Hospital of Xi’an Jiaotong University, Xi’an, 710061 Shaanxi People’s Republic of China; 2 Section of Cancer Stem Cell Research, Key Laboratory of Environment and Genes Related to Diseases, Ministry of Education of the People’s Republic of China, Xi’an, 710061 Shaanxi People’s Republic of China

**Keywords:** Cervical cancer, Mechanisms of disease

## Abstract

HOXA5 is considered a regulator involved in embryonic development and cellular differentiation and a tumor suppressor. Nevertheless, its biological role in cervical carcinoma is still unclear. In the present study, immunohistochemistry showed that HOXA5 expression gradually decreased as the degree of cervical lesions deepened. Ectopic expression of HOXA5 restrained cell proliferation, decreased cell viability, and inhibited tumor formation in vitro and in vivo. Furthermore, the expression of HOXA5 could arrest cell cycle from G0/G1 to S phase. RNA-seq revealed that p21 and cyclinD1 were involved in this process. Moreover, the gene set enrichment analysis and the TOP/FOP reporter assay both suggested that HOXA5 could restrain the activity of the Wnt/β-catenin pathway. Further study using dual-luciferase reporter assay and quantitative chromatin immunoprecipitation assay demonstrated that HOXA5 could directly bind to the TAAT motif within the promoter of TP53 by its HD domain and transactivate TP53, which can upregulate p21. Altogether, our data suggest that HOXA5 inhibits the proliferation and neoplasia via repression activity of the Wnt/β-catenin pathway and transactivating TP53 in cervical cancer.

## Introduction

Cervical carcinoma ranked 4th in both morbidity and mortality in all cancer types in females globally^1^. According to the latest cancer statistics, there were over 569,000 new cervical cancer cases and 311,000 deaths in 2018^1^. The main cause of cervical cancer is considered to be human papillomavirus (HPV) infection^2,3^, but not every patient with infection of HPV develop cervical cancers, only a small proportion of people develop cervical cancer. This phenomenon indicates that there are other elements participating in cervical carcinogenesis^4^. Recent studies indicated stem cell-related genes are related with the development of cervical cancer^5,6^. Previous studies in our laboratory demonstrated that dysfunction of stem cell-related genes, such as SOX17^7^, SLUG^8^, KLF4^9^, GDF15^10^, DAX1^11^, and EZH2^12^ may participate in cervical carcinogenesis. Therefore, there is a compelling need to explore the underlying mechanism of stem cell-related genes in cervical cancer.

HOXA5 is a stem cell-related gene and is a member of homeobox gene clusters. HOXA5 is an important development regulator gene that participates in the development of the respiratory system^13–15^. HOXA5 is also considered a marker of terminal differentiation that exerts mutual antagonism with Wnt signaling^16^. Furthermore, HOXA5 was widely studied in esophageal cancer^17^, gastric cancer^18^, renal cancer^19^, melanoma^20^, breast cancer^21^, and colorectal cancer^16^. To the best of our knowledge, the molecular mechanisms of HOXA5 in the development of cervical carcinoma are mostly unclear.

In the present study, we demonstrate that HOXA5 is deregulated in cervical lesions. Ectopic expression of HOXA5 suppresses the proliferation and neoplasia of cervical cancer cells via repressing the activity of the Wnt/β-catenin pathway and transactivating TP53.

## Materials and methods

### Clinical samples

A total of 42 cervical cancer (CC), 28 high-grade squamous intraepithelial lesion (HSIL), and 55 normal cervix (NC) tissues were gleaned from NO.215 Hospital of Shaanxi Nuclear Industry from January 2015 to December 2018. These patients did not receive immunotherapy, chemotherapy, or radiotherapy. The histological classifications and clinical stage were performed on the basis of FIGO.

### Immunohistochemistry and immunocytochemistry

The technical process of immunohistochemistry (IHC) was performed as previously described^7^. The HOXA5 staining strength was categorized into different groups depending on the positive cell percentage and positive cell staining density. The positive cell percentage was categorized into five grades: 0–3% (0), 3–25% (1), 26–50% (2), 51–75% (3), and 76–100% (4). The positive cell staining density was also categorized into four grades: negative (0), weak brown (1), moderate brown (2), and strong brown (3). The final immunohistochemical score was calculated as follows: immunoreactivity score (IRS) = intensity score × positive score. The final IRS were categorized into three groups: negative (≤3), weak positive (>3 but ≤6), and strong positive (>6). The antibodies used were as follows: anti-HOXA5 (1:100, sc-365784, Santa Cruz); anti-Ki67 (1:100, sc-23900, Santa Cruz); anti-cyclinD1 (1:100, sc-8396, Santa Cruz); anti-p21 (1:50, sc-817, Santa Cruz); anti-β-catenin (1:50, sc-7963, Santa Cruz); anti- p53 (1:50, sc-7963, Santa Cruz). The technical process of immunocytochemistry was performed as previously described^22^.

### Western blot

Fresh tissues and adherent cells were lysed with lysis buffer (the composition of lysis buffer was described previously^7^), which included protease inhibitor cocktail (Roche Diagnostics, USA) for 30 min on ice. After centrifugation, the protein was quantified with BCA quantification (catalogue: 23225, Pierce, USA). Fifty micrograms of cell lysates or tissue lysates were added into sodium dodecyl sulfate-polyacrylamide gel electrophoresis to separate proteins, and then transferred onto the polyvinylidene difluoride (PVDF) membranes. The PVDF membranes were blocked using 5% non-fat milk for 1 h. Then the primary antibodies diluted with 5% non-fat milk were incubated with the membranes overnight at 4 °C. The immunoblot bands were visualized by incubating with horseradish peroxidase (HRP)-conjugated secondary antibodies (Thermo Fisher Scientific). The immunoblot bands were detected with enhanced chemiluminescence reagent (Millipore, Billerica, MA, USA). The primary antibodies used were as follows: anti-HOXA5 (1:1000, sc-365784, Santa Cruz); anti-GAPDH (1:1000, sc-47724, Santa Cruz), anti-β-catenin (1:500, sc-7963, Santa Cruz), anti-c-Myc (1:500, sc-40, Santa Cruz), anti-cyclinD1 (1:1000, sc-8396, Santa Cruz), anti-p53 (1:500, sc-7963, Santa Cruz), and anti-p21(1:1000, #2947, Cell Signaling Technology). The secondary antibodies used for western blot were HRP-conjugated anti-mouse IgG and anti-rabbit IgG (Thermo Fisher Scientific).

### Cell lines and cell culture

The human cervical cancer cell lines (HeLa, SiHa, C-33A, CaSki, HT-3) were obtained from the American Type Culture Collection (ATCC, Rockville, MD, USA). HeLa, SiHa, and C-33A cells were cultured in high-glucose Dulbecco Modified Eagle Medium (Sigma-Aldrich, St. Louis, MO, USA). RPMI1640 Medium (Sigma-Aldrich, St. Louis, MO, USA) was used to culture CaSki cells. McCoy’s 5A Medium (Sigma-Aldrich, St. Louis, MO, USA) was used to culture HT-3 cells. All the cell lines were cultured with the condition in 5% CO_2_ at 37 °C.

### Cell growth and cell viability assays

Cells in the logarithmic growth phase were harvested and counted under aseptic conditions. Cells were inoculated into 35 mm small dishes at 2 × 10^4^ per dish, and cultured with complete medium. When the cells grow to 1, 3, 5, and 7 days, cell numbers were counted. The cell growth curve was generated according to the counting result. The cell viability assays were performed using 3-(4,5-dimethylthiazole-yl)-2,5-diphenyl tetrazolium bromide (MTT, Sigma-Aldrich). At a wavelength of 490 mm, the absorbance was measured using a microplate reader (Bio-Rad, CA, USA). All the experiments were repeated three times independently.

### Tumor xenograft assay

Four to five-week-old female nude mice (BALB/c) were purchased from SLAC Laboratory Animal Co., Ltd. (Shanghai, China). The mice were divided randomly. The mice were bred in a specific pathogen-free condition in which the temperature is at 22–25 °C and the humidity is at 40–50%. The cells in the logarithmic growth phase were counted under sterile conditions. The cell suspension (5 × 10^5^) was injected subcutaneously on the back of nude mice, and the tumor volume was measured every 2–3 days. When the nude mice are sacrificed, the tumor tissue is stripped. The experiment was approved by the Animal Ethics Committee of Xi’an Jiaotong University.

### Flow cytometry analysis

HOXA5-modified cells (4 × 10^5^) were inoculated into 35 mm dishes. After 24 h, the cells were digested with trypsin and fixed with pre-chilled 75% ethanol. After washing twice with phosphate-buffered saline (PBS), the cells were resuspended with 200 μl PBS, and added with 10 μl propidium iodide (1 mg/ml, Sigma Aldrich, St. Louis, MO, USA) and 20 μl RNaseA (1 mg/ml, Sigma Aldrich, St. Louis, MO, USA), and incubate for 35 min on ice. FACS Calibur (BD Biosciences, San Jose, CA, USA) was used for flow cytometer, and the data were analyzed by Flow Jo.

### RNA isolation and quantitative RT-PCR (qRT-PCR)

Total RNA was isolated from cells using RNAiso reagent (Takara, Osaka, Japan), and cDNA was reverse transcripted using PrimeScript RT reagent Kit (Takara, Osaka, Japan). The real-time PCR amplification using Total cDNA as templates, and the SYBR Premix ExTaq II (Takara, Osaka, Japan) was used for real-time quantitative PCR. GAPDH was used as an internal control. Real-time quantitative PCR was performed in triplicate for each sample using TianLong TL988 System (TianLong, Xi’an, China). Results were analyzed using the MED-TL-4CH software. The primers used for real-time quantitative PCR are listed in Table S2.

### RNA preparation and RNA-seq

The RNA of three SiHa-GFP and three SiHa-HOXA5 cell lines were isolated using RNAiso (Takara, Osaka, Japan), and all RNA samples meet a criterion to quality control. The RNA-seq was conducted using the BGISEQ-500 platform. The average output data of each sample was 23.98 Mb. The clean reads of Q30 of the RNA samples were all over 90%. The average contrast ratio of sample to genome was 94.02%, and the contrast ratio of samples to gene set was 83.37%. 17,901 genes were detected. The data was analyzed using NOISeq method with the threshold log2 fold-change >2.

### Plasmids and cell transfection

The CDS of HOXA5 was cloned by PCR and were inserted into pIRES2-AcGFP (Clontech, Mountain View, CA) to construct pIRES2-AcGFP-HOXA5 expressing plasmids. HOXA5-specific shRNAs were cloned and inserted into pGPU6/GFP/Neo (GenePharma, Shanghai, China) to restrain the expression of HOXA5.

The pSpCas9(BB)-2A-GFP (PX458) plasmid (Plasmid #68370) containing SpCas9 and pSpCas9(BB)-2A-Puro (PX459) plasmid (Plasmid #48139) were purchased from Addgene (Cambridge, MA, USA). The single guide RNA targeting the first exon of HOXA5 were designed using the website (http://crispr.mit.edu/).

To obtain the stable transfection cell lines, the plasmids were transfected into cervical cancer cell lines using Lipofectamine 2000 reagent (Invitrogen, Carlsbad, CA, USA) according to the manufacturer’s instructions. G418 (MCE, New Jersey, CA, USA) was added into the media of transfected cells for selection with stress.

### Luciferase reporter assay

The fragments of TP53 promoter were amplified and cloned into pGL3.0-Basic Vector (Promega, Madison, WI, USA) for the luciferase reporter plasmids. The fragments were verified using Sanger sequencing. HOXA5-modified cells were seed into 24-well-plate (8 × 10^5^) and co-transfected with above plasmids and pRL-TK plasmids (Promega) using Lipofectamine 2000 (Invitrogen). Cells were collected 48 h post-transfection, and luciferase activities were detected by luminometer (Promega) using the Dual-Luciferase Assay Kit (Promega, Madison, WI). Data were analyzed as relative luciferase activity (Firefly luciferase activity/Renilla luciferase activity). Each experiment was performed in triplicate.

### Quantitative chromatin immunoprecipitation

The ChIP assay was used to evaluate transcription factor HOXA5 binding sites using the EZ-Magna ChIP Assay Kit (Millipore, Darmstadt, Germany). The protocol was performed as described previously^8^. The chromatin–protein complexes were immunoprecipitated with 10 μg of anti-HOXA5 antibody (sc-365784, Santa Cruz, CA, USA). The DNA fragments obtained from that were then used as templates for real-time PCR. Each sample was performed in triplicate, and the fold enrichment ratio was measured as the value of the ChIP sample vs. the corresponding input sample.

### Statistical analysis

Statistical analysis was conducted using SPSS 19.0 software (SPSS Inc., Chicago, IL). All data are shown as means ± standard deviation of the mean (SD). Gene expression in tumor tissues and their controls were compared by the unpaired *t*-test, and paired samples were compared by the paired *t*-test. In all tests, *p* < 0.05 is regarded as statistically significant.

## Results

### HOXA5 is frequently downregulated in cervical cancers

**Fig. 1:**
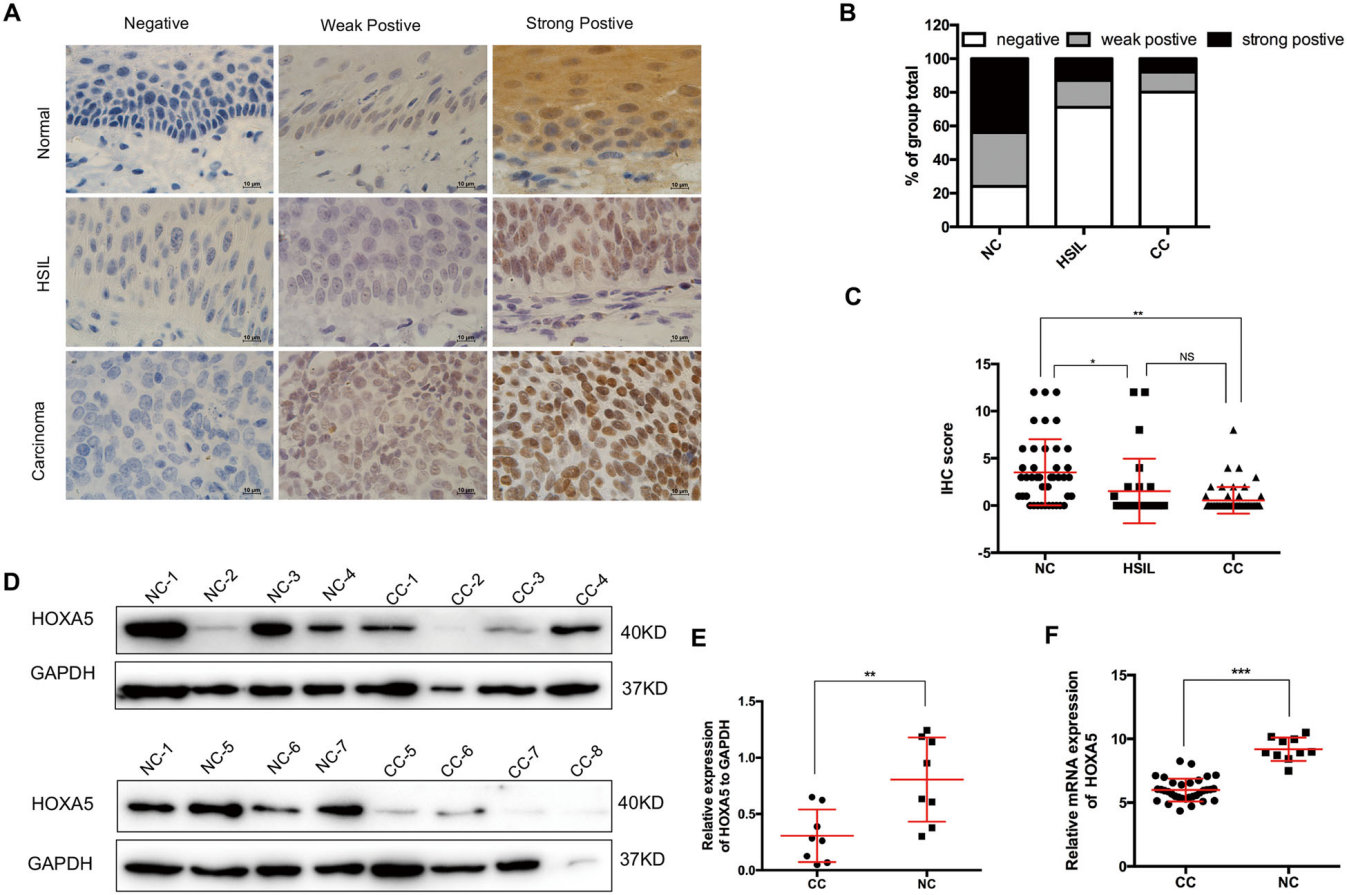
The expression of HOXA5 is down-regulated in cervical carcinomas. **a** Immunohistochemical staining of HOXA5 in clinical samples, including normal cervix (NC, *n* = 42), high-grade squamous intraepithelial lesion (HSIL, *n* = 28), and cervical carcinomas (CC, *n* = 55), original magnification, 1000×. **b** The immunohistochemical staining intensity was classified into negative, weak positive, and strong positive, and the percentage of each group was shown. **c** The scatter plots showed the IHC scores obtained for the staining of HOXA5 in different cervix lesion samples (points represent the IHC score per specimen, Student’s *t*-test is performed). **d** HOXA5 expression was detected by western blot in eight normal cervix samples and eight cervical carcinoma samples. GAPDH was used as loading control. **e** The quantitative illustration of the levels of HOXA5 protein using densitometry to measure the density of the corresponding bands in (**d**). Student’s *t*-test was carried out. **f** The HOXA5 mRNA expression level of clinical cervical cancer samples and normal cervix samples, data were obtained from GEO database. *p < 0.05, **p < 0.01, ***p < 0.001.

To understand the expression pattern of HOXA5 in cervical cancer, HOXA5 protein expression was detected in NC (*n* = 42), HSIL, (*n* = 28), and CC (*n* = 55) by IHC. The results revealed that HOXA5 localized in the nucleus, and representative images are shown in Fig. 1a. The total HOXA5-positive (strongly positive and weakly positive) rate decreased from 76.2% in NC samples to 28.6% in HSIL and then 20.0% in CC samples (Table S1 and Fig. 1b, *p* < 0.05). Significant differences were observed between either of the two groups (Table S1, NC vs. HSIL, *p* < 0.05; NC vs. CC, *p* < 0.001; HSIL vs. CC, *p* < 0.001). Additionally, the analysis of IRS showed that HOXA5 staining was 3.52 ± 0.54 in NC, 1.54 ± 0.65 in HSIL, and 0.56 ± 0.19 in CC (Fig. 1c). Moreover, western blot analysis was performed in eight NC tissue samples and eight CC tissue samples (Fig. 1d). The average HOXA5 expression level in NC (0.8051 ± 0.1321, *n* = 8) was two times higher than that in CC (0.3062 ± 0.08264, *n* = 8) (Fig. 1e). Furthermore, data from the GEO database (GDS2416, GDS3233, and GDS3292) also showed that HOXA5 expression in CC samples was significantly lower than that in NC samples (Fig. 1f). These findings suggest that HOXA5 may play a suppressive role in the carcinogenesis and development of cervical cancer.

### HOXA5 inhibits the proliferation of cervical cancer cells in vitro

**Fig. 2:**
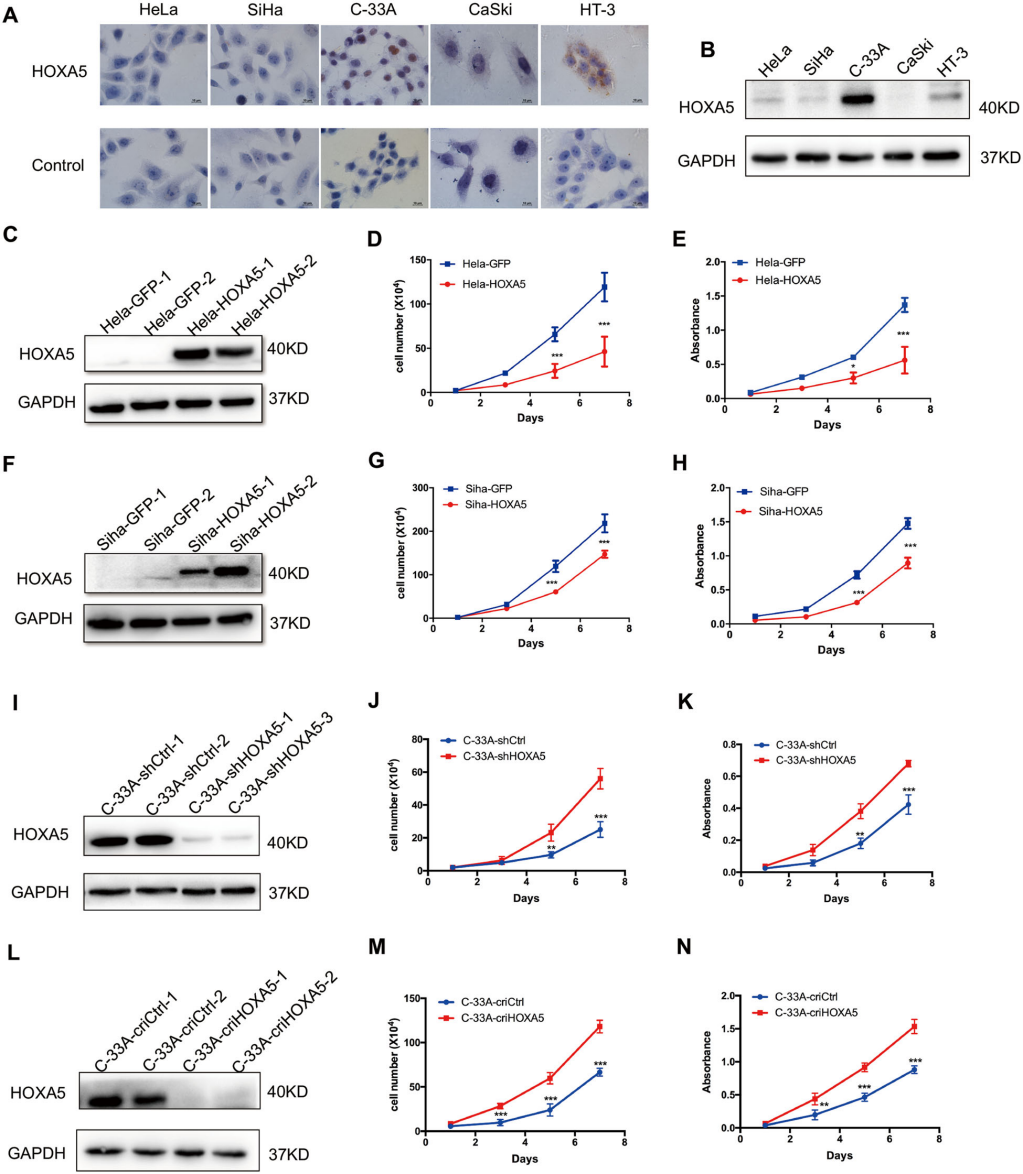
HOXA5 inhibits cervical cancer cell growth in vitro. The expression of HOXA5 in human cervical cancer cell lines was detected using immunocytochemistry (**a**) and western blot (**b**). Stably transfected HOXA5-modified cervical cancer cell lines were identified by western blotting (**c**, **f**, **i**, **l**). The proliferation was detected using growth curves in HeLa-GFP and HeLa-HOXA5 cells (**d**), SiHa-GFP and SiHa-HOXA5 cells (**g**), C-33A-shCtrl and C-33A-shHOXA5 cells (**j**), and C-33A-criCtrl and C-33A-cri HOXA5 cells (**m**). The viability was detected by the MTT assay in HeLa-GFP and HeLa-HOXA5 cells (**e**), SiHa-GFP and SiHa-HOXA5 cells (**h**), C-33A-shCtrl and C-33A-shHOXA5 cells (**k**), and C-33A-criCtrl and C-33A-cri HOXA5 cells (**n**). Data were statistically analyzed with Student’s *t*-test and values are shown as the mean ± SD. *p < 0.05, **p < 0.01, ***p < 0.001.

The expression of HOXA5 protein in cervical cancer cell lines was evaluated by western blot and immunochemistry. As shown in Fig. 2a, b, HOXA5 was highly expressed in C-33A cells, and was expressed to a lower degree in HeLa, SiHa, and HT-3 cells, and almost no expression was detected in CaSki cells. Therefore, HOXA5 was stably overexpressed in HeLa (Fig. 2c) and SiHa cells (Fig. 2f). In addition, HOXA5 was knocked down using small hairpin RNA and knocked out in C-33A cells using CRISPR-Cas9-mediated gene editing (Figs. 2i, l and S1A, B). The overexpression, knockdown, and knockout efficacies of HOXA5 were confirmed by western blot (Fig. 2c, f, i, l).

As shown in Fig. 2d, g, HeLa and SiHa cells overexpressing HOXA5 (HeLa-HOXA5, SiHa-HOXA5) exhibited markedly lower proliferation ability than their control cells (HeLa-GFP, SiHa-GFP, *p* < 0.001). In addition, the viability of HeLa-HOXA5 and SiHa-HOXA5 cells was also significantly lower than that of their control cells (HeLa-GFP, SiHa-GFP, *p* < 0.001). Conversely, knockdown and knockout of HOXA5 in C-33A cells resulted in a remarkable increase in cell proliferation and viability (Fig. 2j, k, m, n, *p* < 0.001). Altogether, these findings demonstrate that HOXA5 inhibits the proliferation of cervical cancer cells in vitro.

### HOXA5 inhibits tumor formation and the proliferation of cervical cancer cells in vivo

**Fig. 3:**
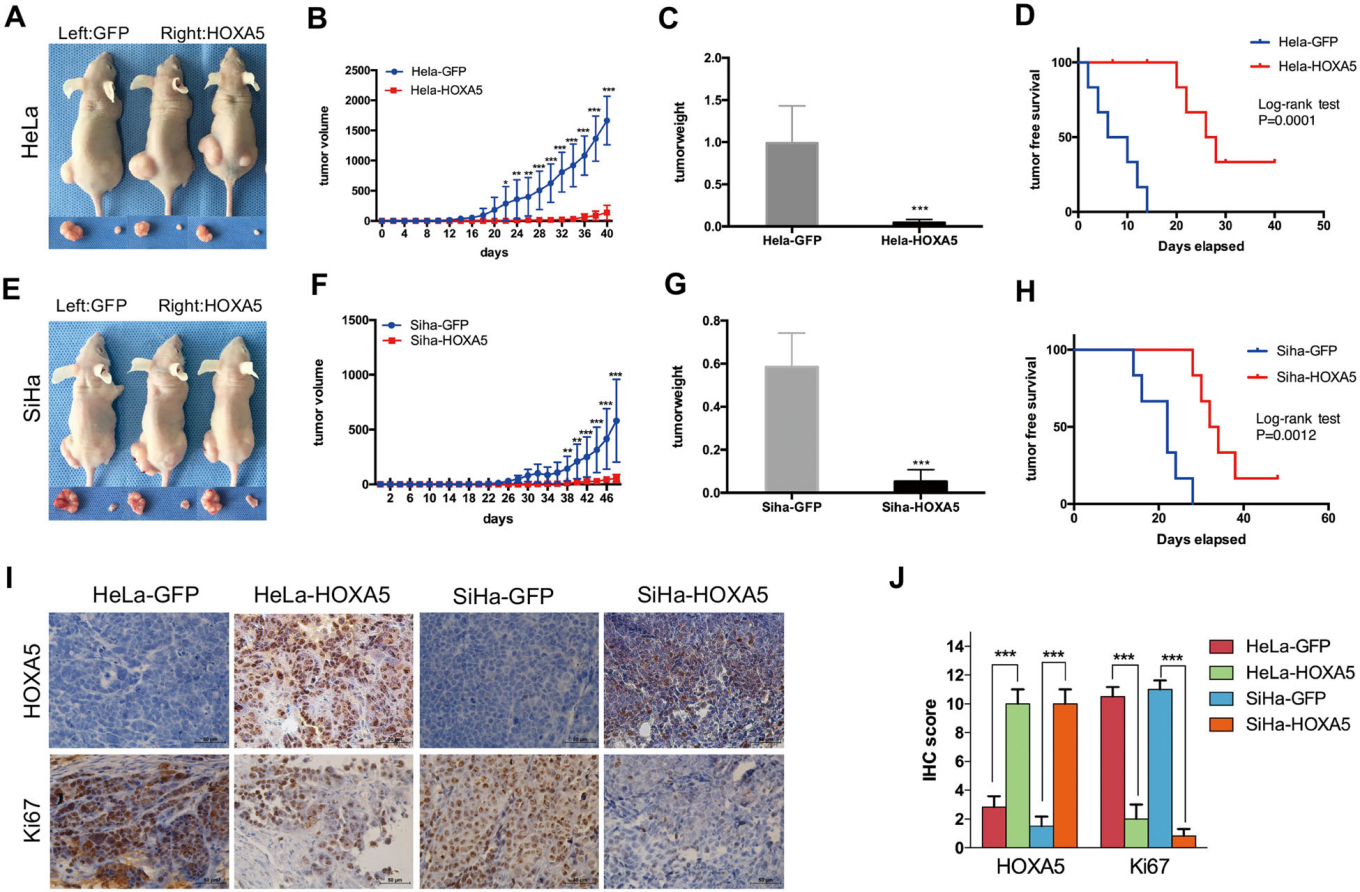
HOXA5 inhibits cervical cancer cell growth in vivo. **a** Xenograft tumor formation of HeLa-GFP and HeLa-HOXA5 cells. The tumor growth curve (**b**), tumor weight (**c**), and tumor-free survival (**d**) of HeLa-GFP and HeLa-HOXA5 cells, respectively. **e** Xenograft tumor formation of SiHa-GFP and SiHa-HOXA5 cells. The tumor growth curve (**f**), tumor weight (**g**), and tumor-free survival (**h**) of SiHa-GFP and SiHa-HOXA5 cells, respectively. **i** Immunohistochemical staining of HOXA5 and Ki67 in xenograft tumor tissues derived from HeLa-GFP cells, HeLa-HOXA5 cells, SiHa-GFP and SiHa-HOXA5 cells, respectively. **j** Immunoreactivity scores of HOXA5 and Ki-67 in xenograft tumor tissues derived from HeLa-GFP cells, HeLa-HOXA5 cells, SiHa-GFP and SiHa-HOXA5 cells. Data were statistically analyzed with Student’s *t*-test and values are shown as mean ± SD. *p < 0.05, **p < 0.01, ***p < 0.001.

To explore the function of HOXA5 in vivo, xenograft assays were performed in nude mice, and the development of solid tumors was monitored. A total of 5 × 10^5^ HOXA5-modified cells (HeLa-HOXA5, SiHa-HOXA5) and their control cells (HeLa-GFP, SiHa-GFP) were injected subcutaneously into the nude mice (Fig. 3a, e). It was obvious that the tumors developed from HeLa-HOXA5 cells grew slower (Fig. 3b, *p* < 0.001) and were lighter (Fig. 3c, 0.9876 ± 0.1811 vs. 0.04345 ± 0.01577, *p* < 0.001; Fig. S2A) than tumors developing from HeLa-GFP cells. Moreover, the survival of free tumors in HOXA5-overexpressing cells was significantly longer than that of their control cells (Fig. 3d, *p* < 0.001). Similar results were obtained in SiHa-HOXA5 cells (Fig. 3f, *p* < 0.001; Fig. 3g, 0.5842 ± 0.06465 vs. 0.05253 ± 0.02261, *p* < 0.001; Fig. 3h, *p* < 0.01; Fig. S2B). These findings demonstrated that HOXA5 could inhibit neoplasia and tumor growth of cervical cancer cells in vivo.

To test whether the tumor suppression function of the HOXA5 protein was due to its ability to suppress cell proliferation, the expression of Ki67, which is an important cell proliferation marker, was evaluated by immunohistochemistry in mice xenograft tissues. Tumor tissue formed by HOXA5-overexpressing cells showed much more HOXA5 staining (Fig. 3i, j, 2.83 ± 0.75 vs. 9.23 ± 1.55, *p* < 0.001; 1.53 ± 0.67 vs. 8.51 ± 1.43, *p* < 0.001) but weaker Ki67 staining (Fig. 3i, j, 10.50 ± 0.67 vs. 2.13 ± 1.10, *p* < 0.001; 11.00 ± 0.63 vs. 0.83 ± 0.48, *p* < 0.001) than those formed by the control cells. These findings suggest that HOXA5 could suppress neoplasia and the development of cervical cancer, and the mechanism of this suppression may be due to its ability to inhibit cell proliferation.

### HOXA5 inhibits the proliferation of cervical cancer cells by arresting the cell cycle process from G0/G1 to S phase

**Fig. 4:**
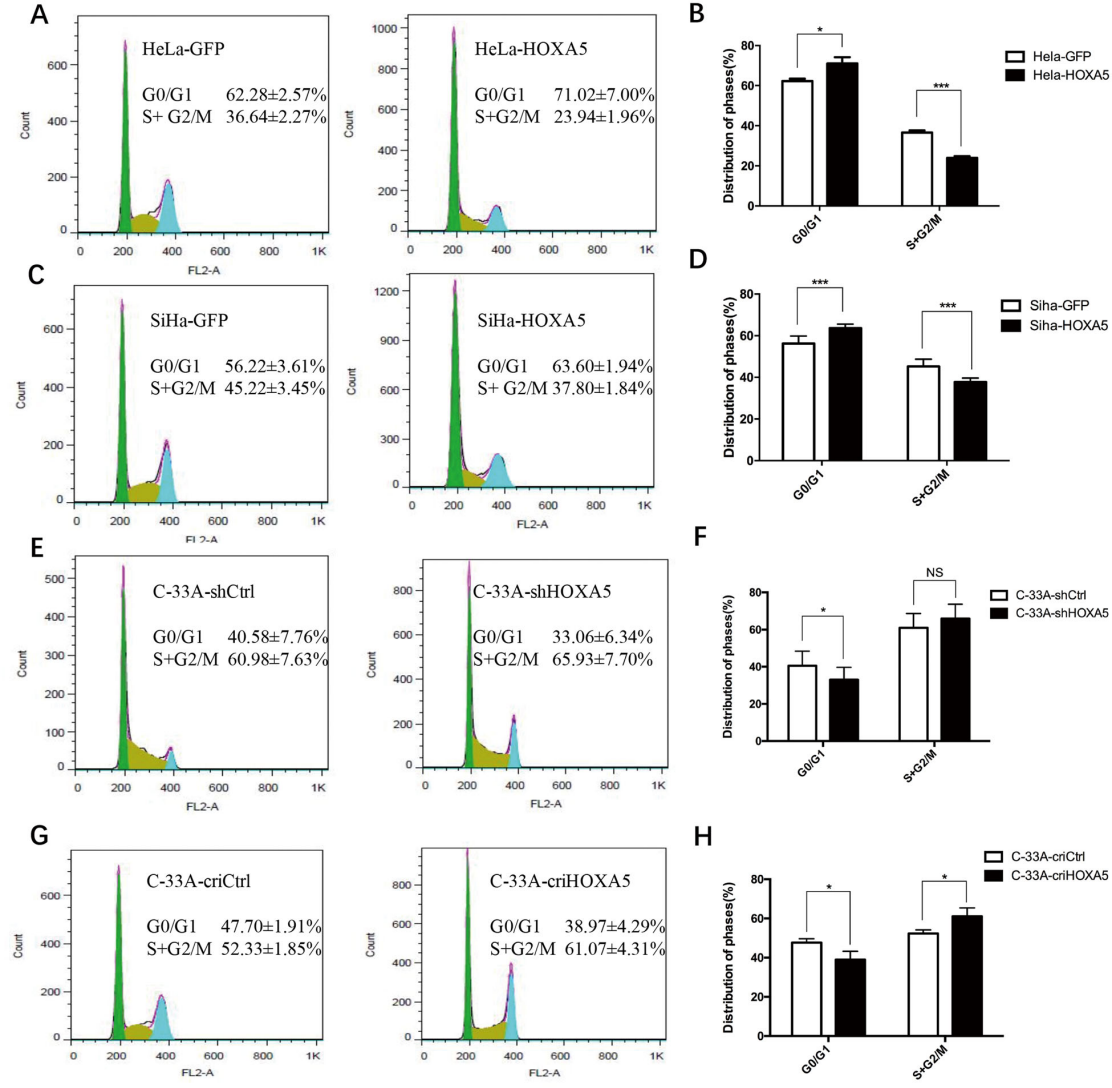
Expression of HOXA5 in cervical cancer cells impeded cell cycle transition from G0/G1 to S phase. **a** In the flow cytometry figures, the *y*-axis shows the count of effective cells and the *x*-axis shows the DNA content. Each colored area represents the cells of different phases of cell cycle: green area refers to the cells in the G0/G1 phase, yellow area refers to the cells in the S phase, and blue area refers to the cells in the G2/M phase. The cell cycles of HeLa-GFP and HeLa-HOXA5 cells were analyzed using flow cytometry (**a**), and a quantitative analysis of the cell cycle are shown (**b**). The cell cycles of SiHa-GFP and SiHa-HOXA5 cells (**c**) and the quantitative analysis (**d**) are shown. The cell cycles of C-33A-shCtrl and C-33A-shHOXA5 cells (**e**) and the quantitative analysis (**f**) are shown. The cell cycles of C-33A-criCtrl and C-33A-cri HOXA5 cells (**g**) and the quantitative analysis (**h**) are shown. The data were shown as the mean ± SD of three independent experiments. Data were statistically analyzed with Student’s *t*-test and values are shown as mean ± SD. *p < 0.05, **p < 0.01, ***p < 0.001.

To understand the mechanism of how HOXA5 protein inhibited the proliferation of cervical cancer cells, fluorescence-activated cell sorting was performed to analyze the cell cycle of HOXA5-modified cells and their control cells. As shown in Fig. 4a, b, ectopic expression of HOXA5 led to a significant increase in the percentage of cells in G0/G1 phase (62.28 ± 2.57% vs. 71.02 ± 7.00%, *p* < 0.05) and a decrease in the percentage of cells in S/G2/M phase (36.64 ± 2.27% vs. 23.94 ± 71.96%, *p* < 0.001). A similar result was obtained in SiHa cells (Fig. 4c, d, 56.22 ± 3.61% vs. 63.60 ± 1.94%, *p* < 0.001; 45.22 ± 3.45% vs. 37.8 ± 1.84%, *p* < 0.001). Conversely, knockdown and knockout of HOXA5 in C-33A cells dramatically reduced the percentage of cells in G0/G1 phase (Fig. 4e–h, 40.58 ± 7.76% vs. 33.06 ± 6.34%, *p* < 0.05; 47.70 ± 1.19% vs. 38.97 ± 4.29%, *p* < 0.05), but increased the percentage of S/G2/M phase cells (Fig. 4e–h, 60.98 ± 7.63% vs. 65.93 ± 7.70%, NS; 52.33 ± 1.85% vs. 61.07 ± 4.31%, *p* < 0.05). These results suggest that HOXA5 inhibits the proliferation of cervical cancer cells by arresting the cell cycle process from G0/G1 to S phase.

### HOXA5 arrests the cell cycle transition from G0/G1 to S phase through cyclinD1 and p21

**Fig. 5:**
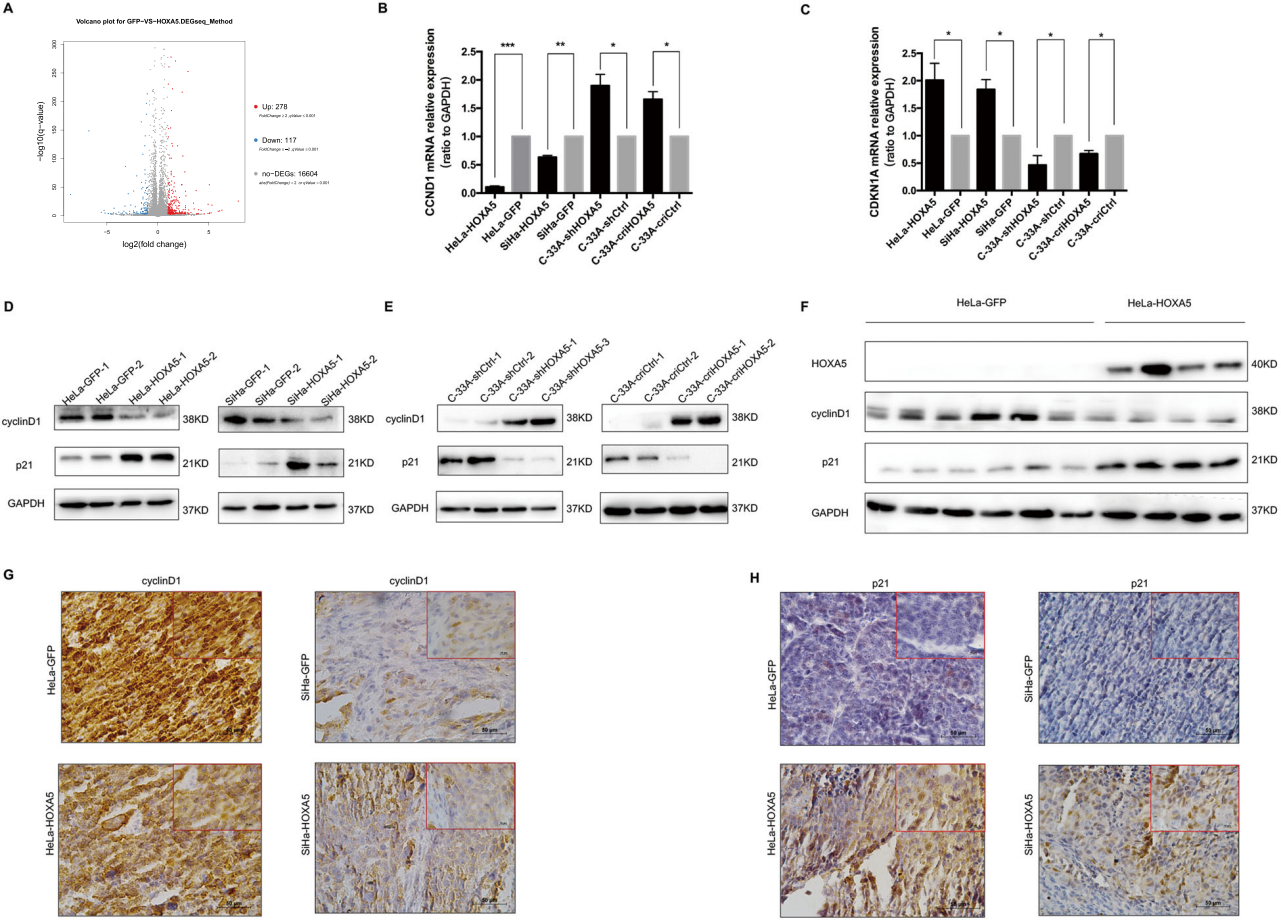
HOXA5 arrests cell cycle transition from G0/G1 to S phase through cyclinD1 and p21. **a** Volcano plots of the data from RNA-seq. The expression of cyclinD1 and p21 in HOXA5-modified cervical cancer cells and xenograft was determined by real-time PCR and western blot (**b**–**e**). The expression of cyclinD1 and p21 in xenograft was determined by western blot (**f**) and IHC (**g**, **h**). *p < 0.05, **p < 0.01, ***p < 0.001.

The cell cycle is regulated by a set of cell cycle-related molecules. To understand HOXA5-induced phenotype changes, we performed RNA-seq (BGI500) in three SiHa-HOXA5 cell lines and three SiHa-GFP cell lines. The results of RNA-seq showed that two cell cycle-related molecules, p21 (the coding gene is called CDKN1A) and cyclinD1 (the coding gene is called CCND1), changed significantly among the 395 most differentially expressed genes (Fig. 5a). As shown in Fig. 5b, c, the mRNA levels of *CCND1* were decreased in HOXA5-overexpressing cells and increased in HOXA5-knockdown and HOXA5-knockout cells. Conversely, the mRNA levels of *CDKN1A* were increased in HOXA5-overexpressing cells and decreased in HOXA5-knockdown and HOXA5-knockout cells (Fig. 5b, c). These data suggested that HOXA5 suppressed the expression of CCND1 and promoted the expression of CDKN1A at the transcriptional level. Consistent with the mRNA results, the p21 protein was significantly increased in HOXA5-overexpressing cells and xenografts derived from HOXA5-overexpressing cells. Conversely, cyclinD1 protein expression was decreased in HeLa-HOXA5 and SiHa-HOXA5 cells and xenografts derived from these cell lines (Fig. 5d–f). The results were also supported by IHC assays (Figs. 5g, h and S3A, B). These data suggested that HOXA5 regulated the expression of cyclinD1 and p21 at the translational level. All the above data demonstrate that HOXA5 possibly arrest the cell cycle process from G0/G1 to S phase through cyclinD1 and p21.

### HOXA5 suppresses the expression of cyclinD1 by inhibiting the activity of the Wnt/β-catenin pathway in cervical cancer cells

**Fig. 6:**
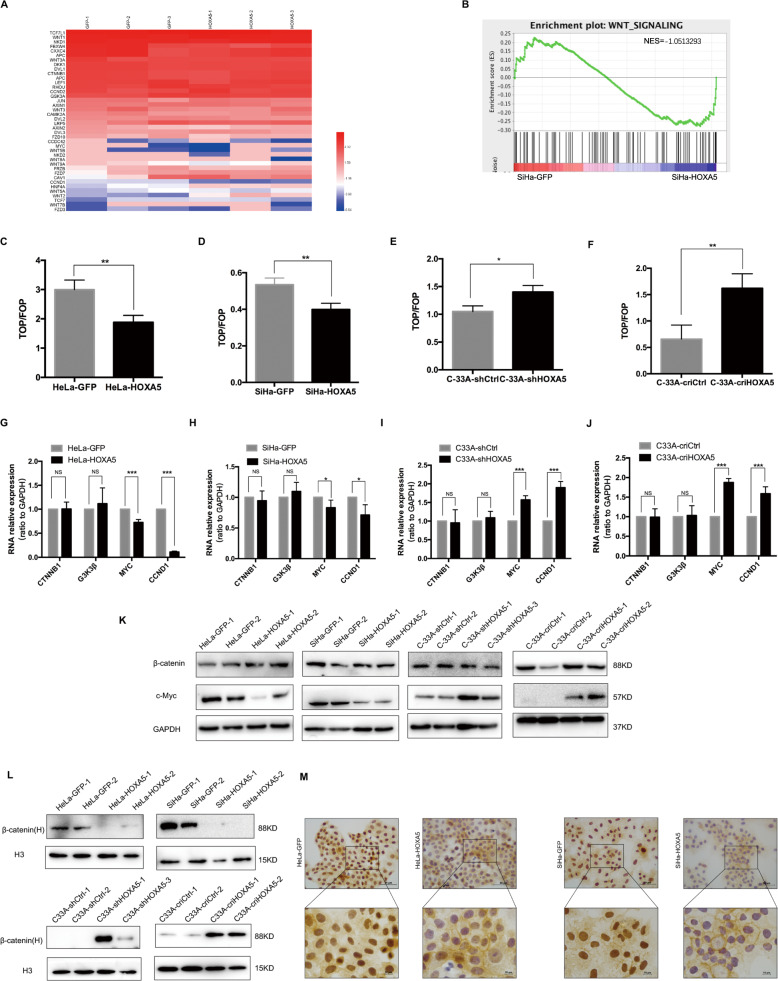
HOXA5 inhibited the expression of cyclinD1 via suppressing the Wnt/β-catenin pathway in cervical cancer cells. **a** Heatmap of known genes for Wnt/β-catenin pathway in SiHa-GFP cells (left) and SiHa-HOXA5 cells (right) using data from RNA-seq. Data were log2 normalized. **b** A gene set enrichment (GSEA) assay using data from RNA-seq. **c**–**f** TOP/FOP-Flash reporter assays were carried out in HOXA5-modified cervical cancer cells. **g**–**j** Real-time PCR analysis was shown for the mRNA levels of Wnt/β-catenin pathway key genes in HOXA5-modified cervical cancer cells. **k** The expression of Wnt/β-catenin pathway key proteins in HOXA5-modified cervical cancer cells was determined by western blot. The nuclear β-catenin of HOXA5-modified cervical cancer cell was determined by western blot (**l**) and immunocytochemistry (**m**). Data represent mean ± SD of triplicate experiments and statistical analysis was done with Student’s *t*-test. *p < 0.05, **p < 0.01, ***p < 0.001.

Ordonez-Moran et al. reported that there is a mutual antagonistic relationship between HOXA5 and the Wnt pathway^16^. Since a dual-luciferase reporter assay showed that HOXA5 did not directly bind to the promoter of CCND1 (Fig. S4A), we hypothesized that the overexpression of HOXA5 could affect the expression of cyclinD1 through the Wnt pathway. Among the changed genes in RNA-seq, we identified 46 genes which are related with Wnt/β-catenin signaling pathway that were differentially expressed (Fig. 6a). A gene set enrichment analysis (GSEA) also indicated that Wnt/β-catenin pathway was repressed in SiHa-HOXA5 cells (Fig. 6b). To further detect the changes of Wnt/β-catenin pathway, the TOP/FOP flash luciferase reporter assays were conducted. Compared with the control cells, ectopic expression of HOXA5 led to a decrease of TOP flash luciferase reporter activity in HeLa and SiHa cells (Fig. 6c, d). However, knockdown and knockout of HOXA5 increased the activity of the TOP flash luciferase reporter in C-33A cells (Fig. 6e, f). Further study demonstrated that the overexpression of HOXA5 repressed the activity of the TOP flash luciferase reporter in a dose-dependent manner (Fig. S4B). These data demonstrated that the activity of Wnt/β-catenin pathway was inhibited by HOXA5 in cervical cancer cell lines. Since the Wnt/β-catenin pathway involves a set of molecules, we detected the mRNA and protein levels of the key molecules of the Wnt/β-catenin signaling pathway CTNNB1, MYC, CCND1, and GSK3β. As Fig. 6g–k shows, the mRNA and protein levels of MYC and CCND1 decreased strongly in HeLa-HOXA5 and SiHa-HOXA5 cells and the xenografts derived from HOXA5-overexpressing cells (Fig. S4C–H). However, the mRNA and protein levels of GSK3β and CTNNB1 did not show any changes after HOXA5 modified. As reported previously, the nuclear accumulation of β-catenin triggered a downstream molecules cascade. To detect the underlying mechanism, we performed a nuclear separation assay on HOXA5-modified cells. Although total β-catenin did not show any changes, the distribution of β-catenin in the nucleus was significantly decreased in HOXA5-overexpressing HeLa and SiHa cells and was significantly increased in HOXA5-knockdown and HOXA5-knockout C-33A cells (Fig. 6l). Immunochemistry also showed the same results (Fig. 6m). All these data indicate that HOXA5 suppressed the expression of cyclinD1 by inhibiting the activity of the Wnt/β-catenin signaling pathway through inhibition of the nuclear translocation of the β-catenin protein in cervical cancer.

### HOXA5 promotes the expression of p21 by transactivating TP53 through direct binding to its promoter in cervical cancer cells

**Fig. 7:**
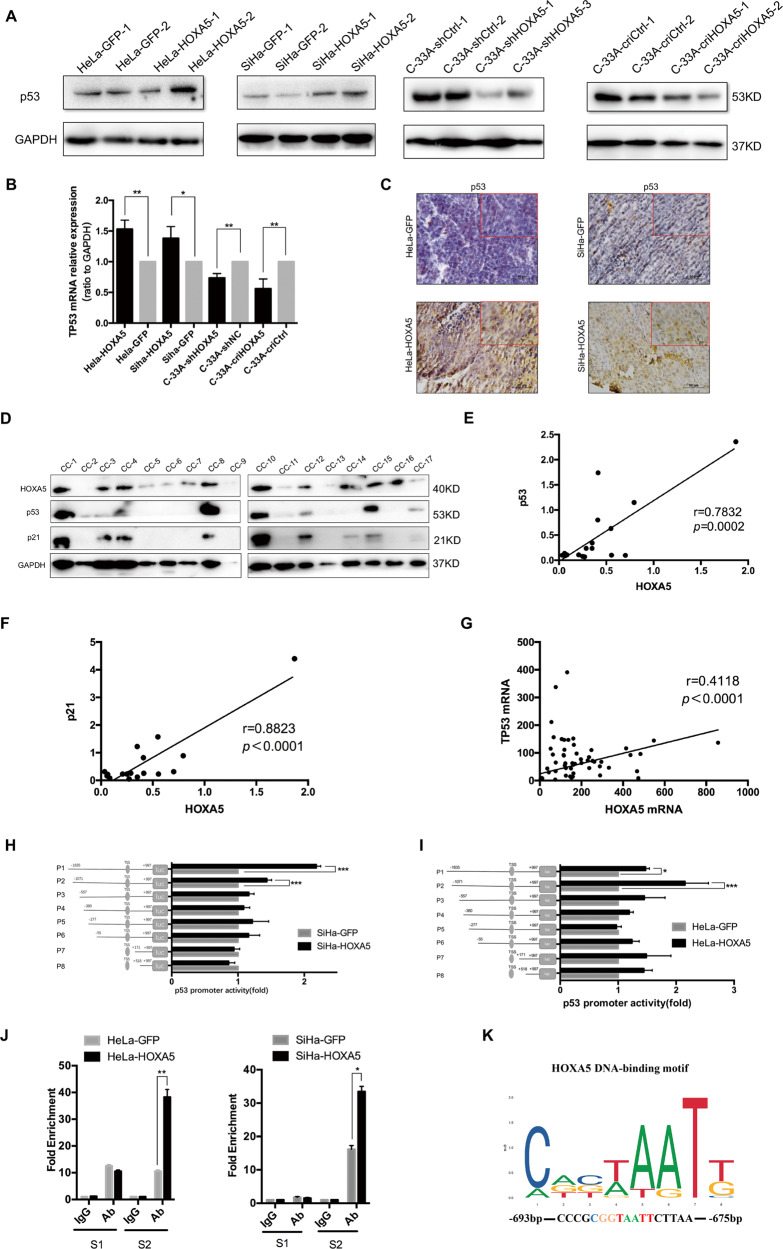
HOXA5 regulates the expression of TP53 in cervical cancer cells. The expression of TP53 in HOXA5-modified cervical cancer cells was determined by real-time PCR (**a**) and western blot (**b**). The expression of p53 in xenograft was determined by IHC (**c**). **d**–**f** The expression of HOXA5, p53, and p21 in clinical cervical cancer specimens was determined by western blot and the correlation was analyzed. **g** HOXA5 expression was significantly positively correlated with the expression of p53 (*r* = 0.4118, *p* < 0.001) in cervical cancers (data from GEO database). **h**, **i** The TP53 promoter structure was constructed and luciferase activity relative to Renilla control was measured in HeLa-HOXA5 and SiHa-HOXA5 cells. **j** The qChIP assay is shown in HeLa-HOXA5 and SiHa-HOXA5 cells immunoprecipitation by HOXA5 antibody and IgG antibody (as the negative control). **k** An experimentally defined transcription factor binding sites of HOXA5 was found in the JASPAR CORE database. The data were shown as the mean ± SD of three independent experiments. *p < 0.05, **p < 0.01, ***p < 0.001.

Since HOXA5 did not bind to the promoter of CDKN1A in HeLa and SiHa cells (Fig. S5A), and p21 is a well-defined downstream factor of p53^23,24^, we speculated that HOXA5 regulated the expression of p21 protein by transactivating TP53. The results showed that p53 was up-regulated in HOXA5-overexpressing cells, while it was down-regulated in HOXA5-knockdown and HOXA5-knockout C-33A cells, at both transcriptional and translational levels (Figs. 7a–c and S5B). To validate the correlation between HOXA5 and p53, the protein levels of HOXA5 and p53 in clinical cervical cancer specimens were detected by western blot (Fig. 7d). The results suggested that p53 and p21 were both positively correlated with HOXA5 (Fig. 7e, *r* = 0.7832, *p* = 0.0002; Fig. 7f, *r* = 0.8823, *p* < 0.0001). Then, the correlation between HOXA5 and p53 in the GEO database (GDS2416, GDS3233, and GDS3292) was analyzed in a large cohort of cervical cancers. As expected, there was a significant positive correlation between the expression of HOXA5 and the expression of p53 (Fig. 7g, *r* = 0.4118, *p* < 0.0001). To further characterize the relationship between HOXA5 and p53, the HOXA5 plasmid was transfected into HeLa parental cells, and the cells were harvested at different times. The results showed that p53 protein gradually increased as time lasted (Fig. S5C, D).

By analyzing the promoter of TP53 on an online database (http://jaspar.genereg.net), the classical TAAT motif (classical homeobox gene binding motif) was found in the promoter of TP53 (Fig. 7k). Therefore, a dual-luciferase reporter assay was used to determine whether HOXA5 directly binds to the promoter of TP53. We cloned the full-length TP53 promoter (−1835/+997 bp) into a pGL3.0-basic luciferase plasmid (pGL3.0-TP53). Transient expression of HOXA5 by the transfection of the luciferase construct into 293T cells resulted in a significant induction of luciferase activity driven by the TP53 promoter compared with the control plasmid (Fig. S5E). To explore the core binding region within the TP53 promoter, the promoter of TP53 was divided into eight different lengths, each of which contained HOXA5 binding motifs (P1: −1835/+997; P2: −1071/+997; P3: −557/+997; P4: −380/+997; P5: −277/+997; P6: −55/+997; P7: +171/+997; and P8: +518/+997). The pGL3.0-basic luciferase plasmid containing different lengths of the TP53 promoter was then transfected into HOXA5-modified cells. The results showed that pGL3.0-TP53-P1 and pGL3.0-TP53-P2 exhibited a significant increase in luciferase activity in HeLa-HOXA5 and SiHa-HOXA5 cells compared with their control cells (Fig. 7h, i). These results suggested that the sequence between nucleotides −1071 and −557 in the promoter of TP53 may contain HOXA5 binding sites.

To further determine the direct regulation of TP53 by HOXA5 through binding to its promoter, a ChIP assay was performed in HOXA5-modified cells. RT-PCR applying specific primers to S1 (−1071 to −712) and S2 (−711 to −581) was conducted to amplify the binding region. The results suggested that HOXA5 strongly bound to S2 in the TP53 promoter region in both HeLa and SiHa HOXA5-overexpressing cells (Fig. 7j). In accordance with the analysis in the JASPAR CORE database, 5′-TAAT-3′ was located between nucleotides −693 and −675 in S2 of the P2 promoter (Fig. 7k).

Among the HOXA5 binding motifs, 5′-TAAT-3′ is the most important. To further evaluate the importance of this motif, a mutant pGL3.0-TP53 plasmid with mutated 5′-TAAT-3′ to 5′-TAAG-3′ was constructed (pGL3.0-TP53-P2-mut). A decrease in TP53 promoter activity was shown after the transient expression of HOXA5 by the transfection of the mutant luciferase construct in 293T cells (Fig. S5F). It has been reported that the HOX family expresses a conserved HD domain that functions in binding DNA^25^. Therefore, an HD domain-deleted mutant of the HOXA5 plasmid (HOXA5-ΔHD) was constructed. The co-transfection of the HOXA5-ΔHD plasmid with the pGL3.0-TP53 plasmid failed to show an increase in luciferase activity (Fig. S5G). The study by Teo and colleagues also obtained the same result in breast cancer cell lines^21^. These data demonstrated that HOXA5 promoted the expression of the p21 protein by directly binding to the 5′-TAAT-3′ motif in the promoter of TP53 through its HD domain in cervical cancer cells.

### Upregulating β-catenin or inhibiting p53 only partly rescues the proliferation inhibitory effect of HOXA5

The results above suggested that the suppressed function of HOXA5 was mediated by cyclinD1 and p21, but it is unclear whether there is crosstalk between the Wnt pathway and p53. Thus, we tested the effect of activating the Wnt/β-catenin pathway in HOXA5-modified cervical cancer cells via transient transfection of the β-catenin plasmid. The results showed that the proliferation-inhibiting ability of HOXA5 in cervical cancer cells was partly abolished upon overexpression of β-catenin in both HOXA5-overexpressing HeLa and SiHa cells (Fig. S6A, B). Moreover, we also found that the overexpression of β-catenin reversed the inhibitory effect of HOXA5 on c-Myc and cyclinD1 proteins but did not affect the protein levels of p21 and p53 (Fig. S6C). Furthermore, the specific p53 inhibitor Pifithrin-α (PFTα) inhibited the expression of p21, as well as p53 protein, and partly rescued the proliferative ability of HOXA5-overexpressing HeLa and SiHa cells. (Fig. S6D, E). However, the proteins in the Wnt/β-catenin pathway were not affected by p53 inhibition (Fig. S6F). All these data suggested that there is possibly no crosstalk between the Wnt pathway and p53.

### Correlation analysis of the expression of HOXA5 with the Wnt pathway and p53 in human cervical cancer tissues

**Fig. 8:**
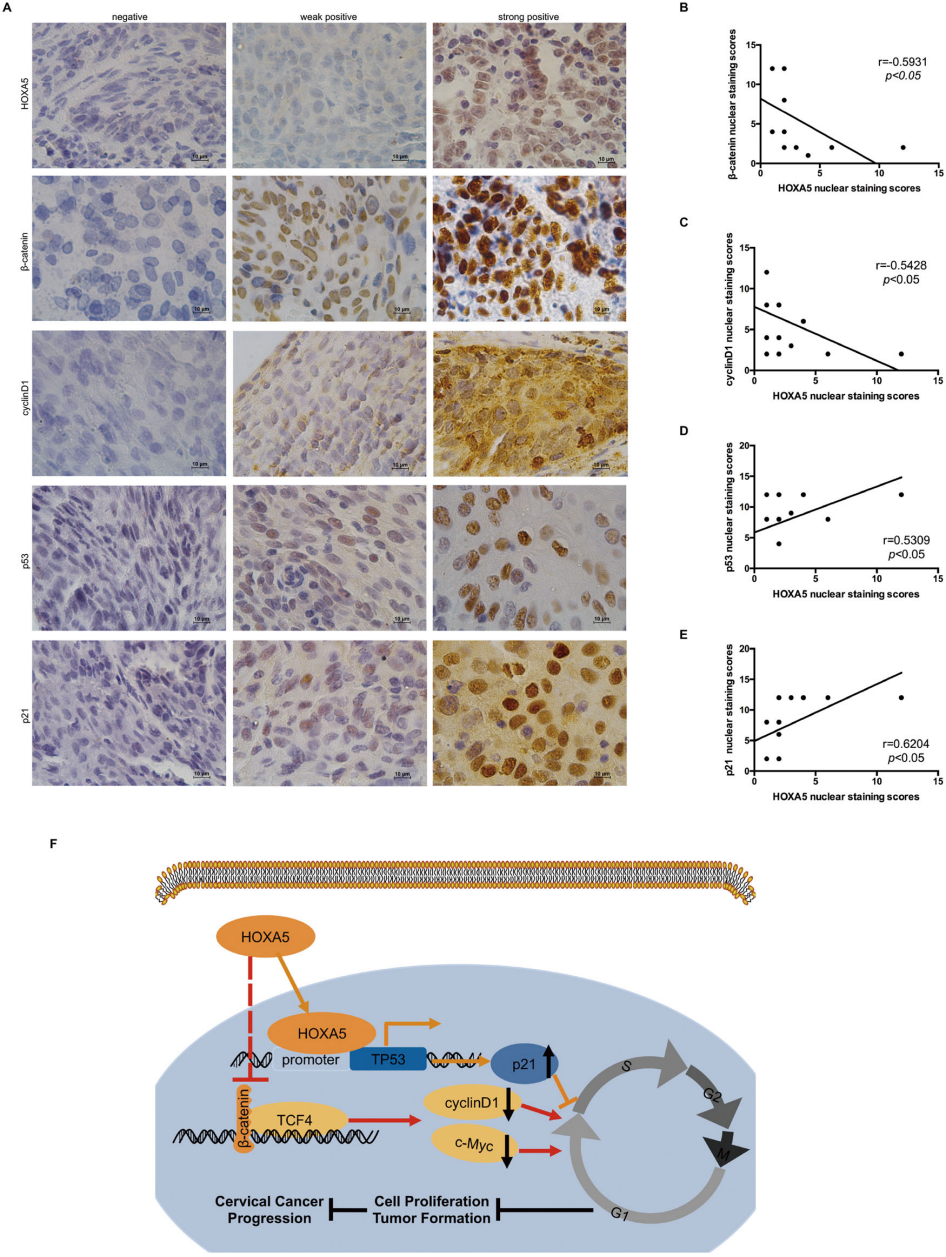
Correlation analysis between the expression of HOXA5 with Wnt signaling and p53 in human cervical cancer specimens. **a** Fifteen cervical cancer specimens were analyzed by IHC, and the representative images of HOXA5, β-catenin, cyclinD1, p53, and p21 were shown. **b**–**e** Correlation of the HOXA5 staining with β-catenin (*r* = 0.5931, *p* < 0.05), c-Myc (*r* = 0.5186, *p* < 0.05), cyclinD1 (*r* = 0.5428, *p* < 0.05), TP53 (*r* = 0. 5309, *p* < 0.05), P21 (*r* = 0.6204, *p* < 0.05). **f** The proposed mechanism of HOXA5 in cervical cancer cells.

To further confirm the relationship between HOXA5 and the proteins in Wnt/β-catenin pathway and p53 in cervical cancer specimens, the expression of HOXA5, β-catenin, cyclinD1, p53, and p21 was detected by IHC in 15 cervical cancer specimens (Fig. 8a). As expected, HOXA5 expression was negatively correlated to the expression of β-catenin and cyclinD1 (Fig. 8b, *r* = −0.5931, *p* < 0.05; Fig. 8c, *r* = −0.5428, *p* < 0.05), while HOXA5 expression was positively correlated with the expression of p53 and p21 (Fig. 8d, *r* = 0.5309, *p* < 0.05; Fig. 8e, *r* = 0.6204, *p* < 0.05). These results implied that HOXA5 possibly affected the proliferation of cervical cancer cells by both the Wnt/β-catenin/cyclinD1 and p53/p21 pathways.

## Discussion

HOXA5 has been reported to be involved in embryo development^26,27^, hematopoietic cell fate^28,29^, and carcinogenesis^30^. To our knowledge, there were several studies concerning the function of HOXA5 protein in cervical carcinoma. An analysis based on the TCGA database demonstrated that the up-regulation of HOXA5 is correlated with poor survival outcomes in cervical cancer^31^. However, Pei and colleagues found that HOXA5 was lowly expressed in cervical squamous cell carcinoma patients with poor differentiation^32^. Another report demonstrated that HOXA5 could suppress the proliferation and invasion and induce apoptosis through AKT and p27 in cervical cancer cells^33^.

Although the incidence of cervical cancer is decreasing globally, it is still the deadliest cancer in Africa and South America^1^. It has been postulated that there is a consecutive progression from LSIL to HSIL and then to cervical carcinoma. In the present research, both the data from clinical specimens and the data from the GEO database indicated that the expression of HOXA5 protein was downregulated in cervical cancer. These results indicate HOXA5 is closely related with the neoplasia and development of cervical carcinoma.

Next, cell growth curves, MTT assays, and tumor xenografts showed that HOXA5 protein could inhibit tumor formation and progression by inhibiting cell proliferation. Furthermore, the cell cycle analysis revealed that HOXA5 could induce cell cycle arrest from G0/G1 phase to S phase. These results demonstrate that HOXA5 inhibited cell proliferation by inducing cell cycle arrest from G0/G1 phase to S phase.

The cell cycle is monitored by a set of cell cycle-related molecules. In different phases, diverse cell cycle proteins show different expression and degradation patterns, helping to coordinate the timing of every mitotic event^34^. In this study, functional experiments suggested that HOXA5 inhibited cell proliferation via inducing the cell cycle arrest from G0/G1 phase to S phase. Our results showed that the expression of HOXA5 could induce obvious changes in cell cycle-related molecules, especially downregulation of cyclinD1 and upregulation of p21.

It has been reported that Wnt/β-catenin signaling is related with the tumorigenesis in cervical carcinoma. Several studies have indicated Wnt/β-catenin is activated in the development of cervical cancer^35^. Positive β-catenin staining was observed in 67.9% of cervical squamous cell carcinoma patients and the expression of it has a positive correlation with poor disease-free survival, as well as poor overall survival^36^. However, the underlying mechanism was not further elucidated. β-Catenin regulates a series of downstream target genes, including c-Myc and cyclinD1, which regulates the cell cycle process^37^. In the present study, the significant decrease in cyclinD1 prompted us to determine whether HOXA5 inhibited the expression of cyclinD1 via the Wnt/β-catenin pathway. Our findings strongly demonstrate that HOXA5 inhibited the expression of cyclinD1 via the Wnt/β-catenin pathway.

p53 is a crucial protein in cervical cancer progression. The HPV E6 oncoprotein can activate several mechanisms to downregulate p53 and interfere with p53 to block the function of the p53 protein^38,39^. Since p53 protein participates in complex biological processes in vivo, it can both induce the anti-proliferation and apoptosis effect in multiple tumors^40^. In the present study, another important cell cycle molecule that showed a significant change was the p21 protein. As reported previously, p21 is a well-defined downstream factor of the p53 pathway^23^. Therefore, we found both the data obtained from our clinical specimens and the data from the GEO database showed a strong correlation between HOXA5 and p53.

To further explore whether there is a direct relationship between HOXA5 and p53, a dual-luciferase assay was conducted. The results suggested HOXA5 could bind to the TAAT motif within the promoter of TP53 by its HD domain directly. The qChIP assay further confirmed that HOXA5 binds between −693 and −675 in the promoter of TP53 and acts as a transcriptional activator to induce the expression of TP53. This is the first study demonstrating that HOXA5 affects the proliferation of cancer cells through both the Wnt/β-catenin/cyclinD1 and p53/p21 signaling pathways simultaneously.

In conclusion, our findings demonstrated that HOXA5 suppressed the activity of the Wnt/β-catenin pathway and transactivated TP53 simultaneously, thus inhibiting the cell cycle progression by downregulating cyclinD1 and upregulating p21, ultimately inhibiting the neoplasia and progression in cervical cancer.

## Supplementary information

Figure S1

Figure S2

Figure S3

Figure S4

Figure S5

Figure S6

Table S1

Table S2

Supplement Figure Legends
