# Supplementary figures and images for: HOXA5 inhibits the proliferation and neoplasia of cervical cancer cells via downregulating the activity of the Wnt/β-catenin pathway and transactivating TP53
Source: Cell Death Dis. 2020 Jun 4;11(6):420. doi: 10.1038/s41419-020-2629-3 (PMC7272418; doi:10.1038/s41419-020-2629-3)

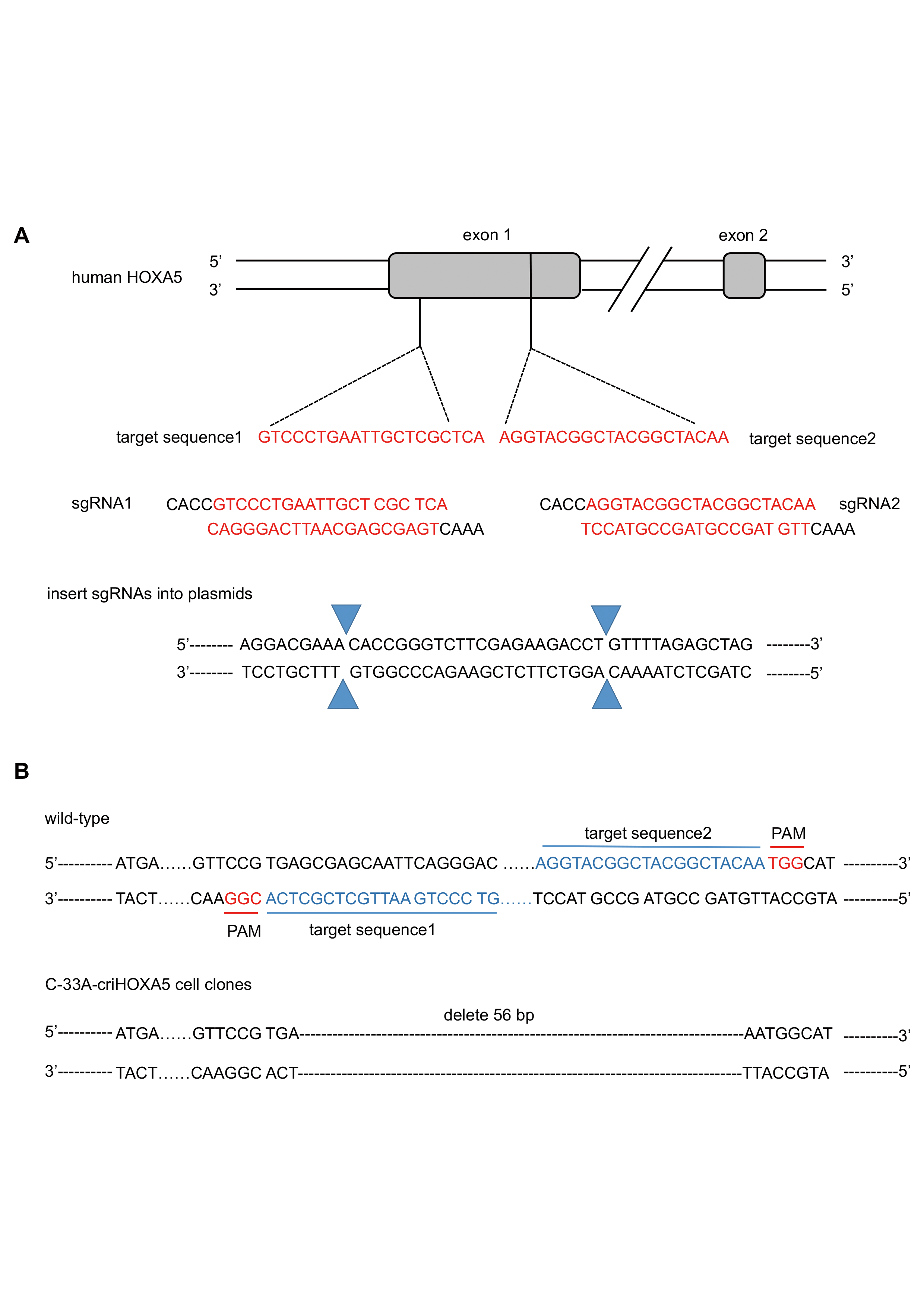

Supplement: Supplementary file 1 — Figure S1 [file 41419_2020_2629_MOESM1_ESM.png]

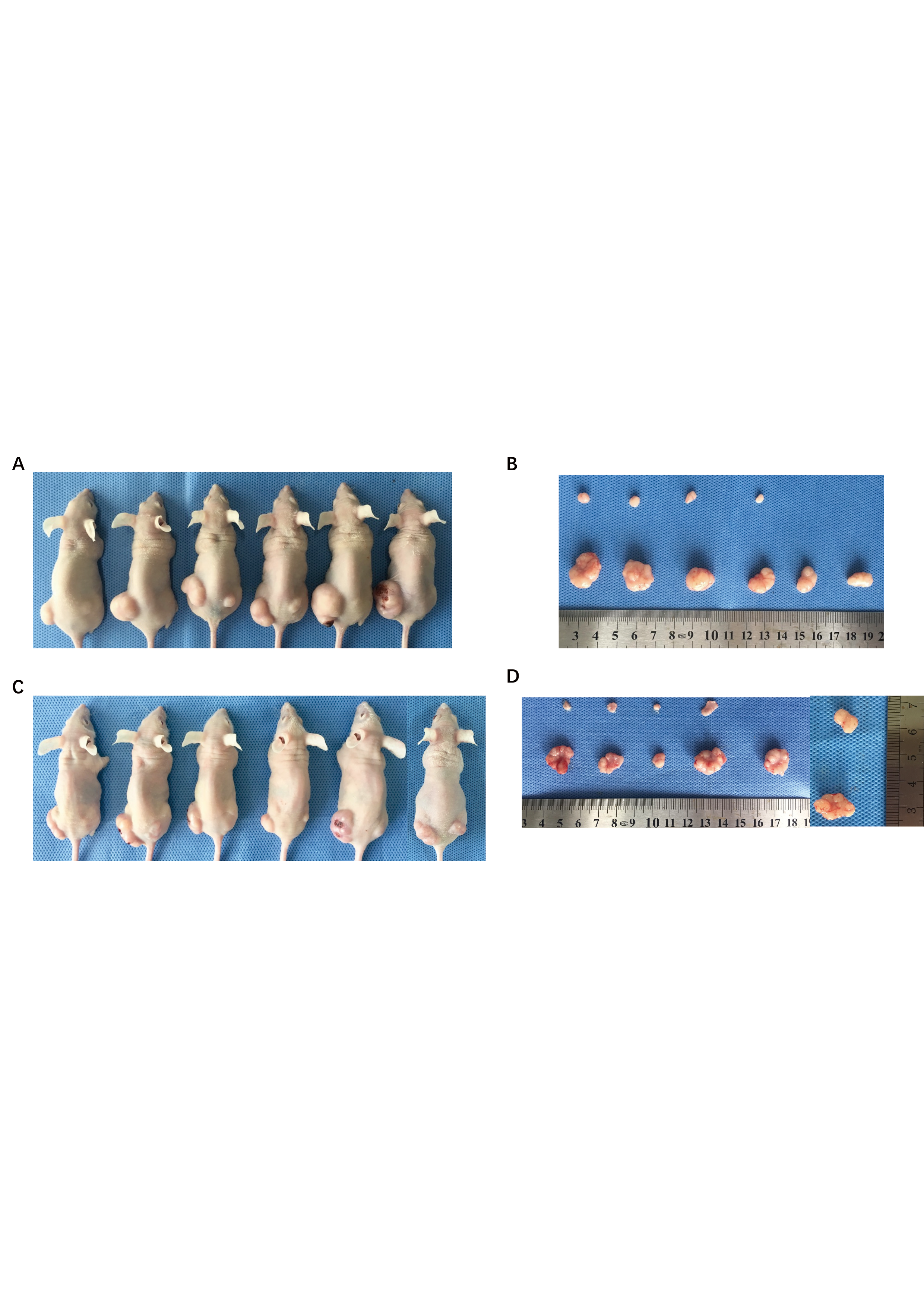

Supplement: Supplementary file 2 — Figure S2 [file 41419_2020_2629_MOESM2_ESM.png]

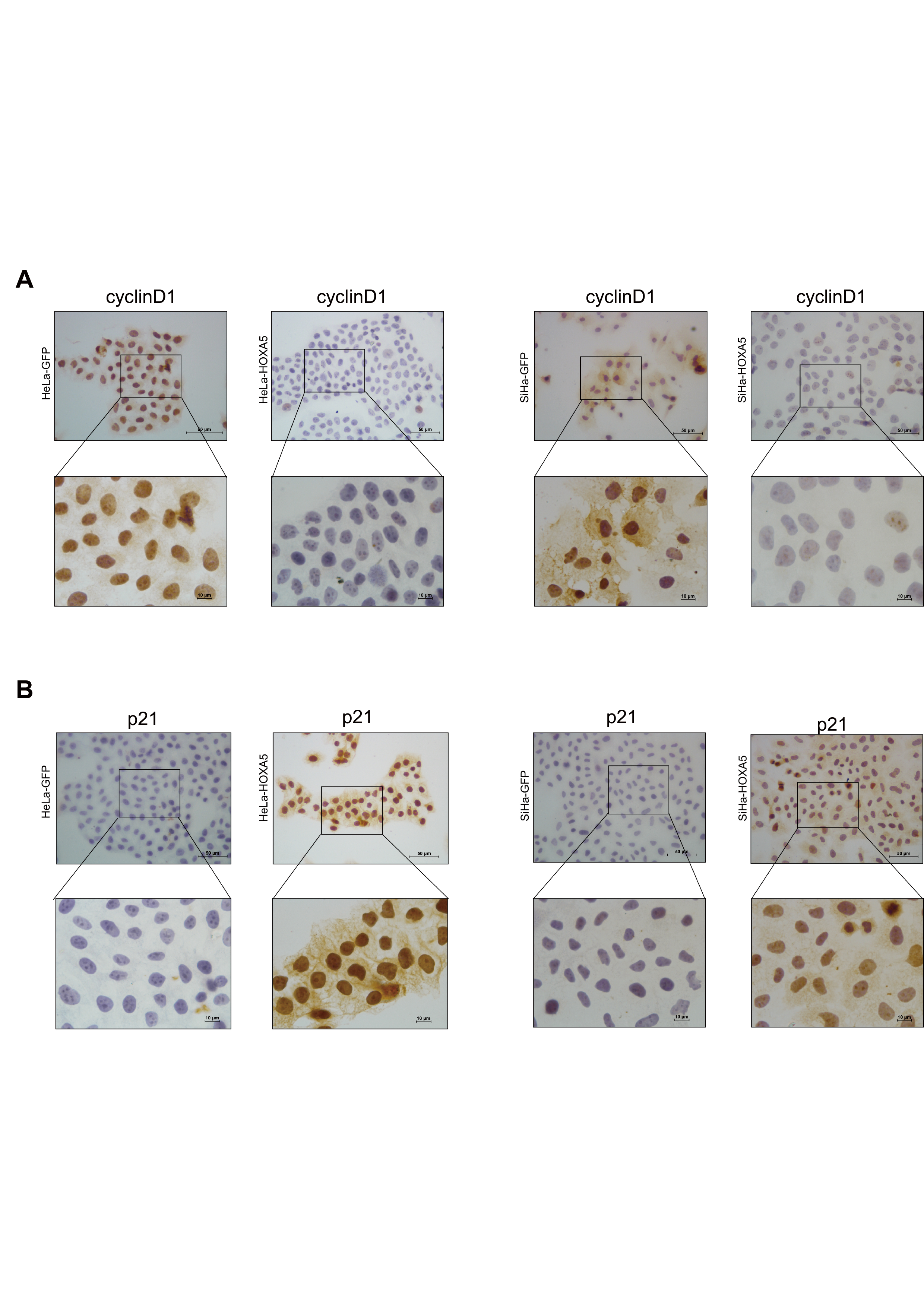

Supplement: Supplementary file 3 — Figure S3 [file 41419_2020_2629_MOESM3_ESM.png]

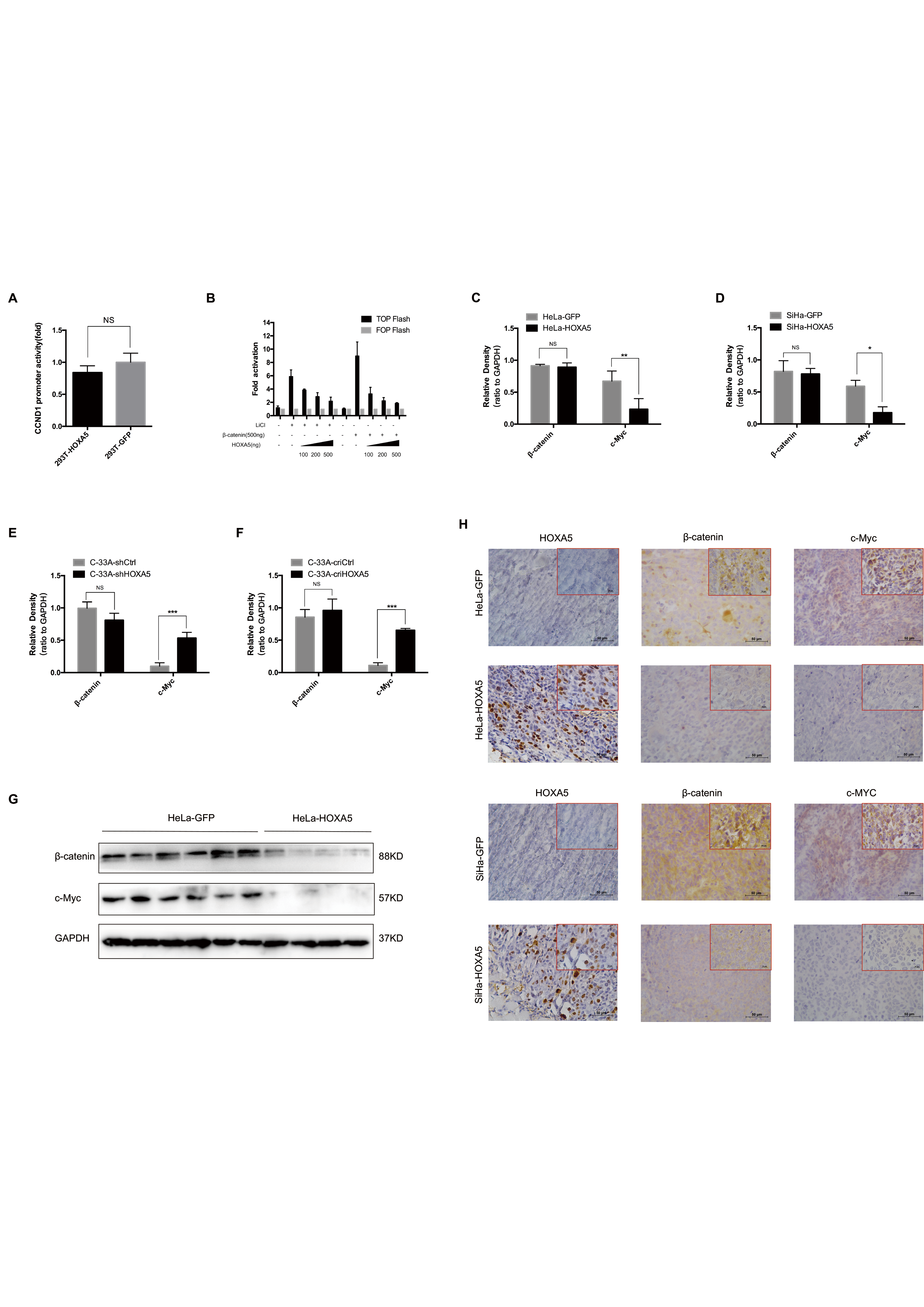

Supplement: Supplementary file 4 — Figure S4 [file 41419_2020_2629_MOESM4_ESM.png]

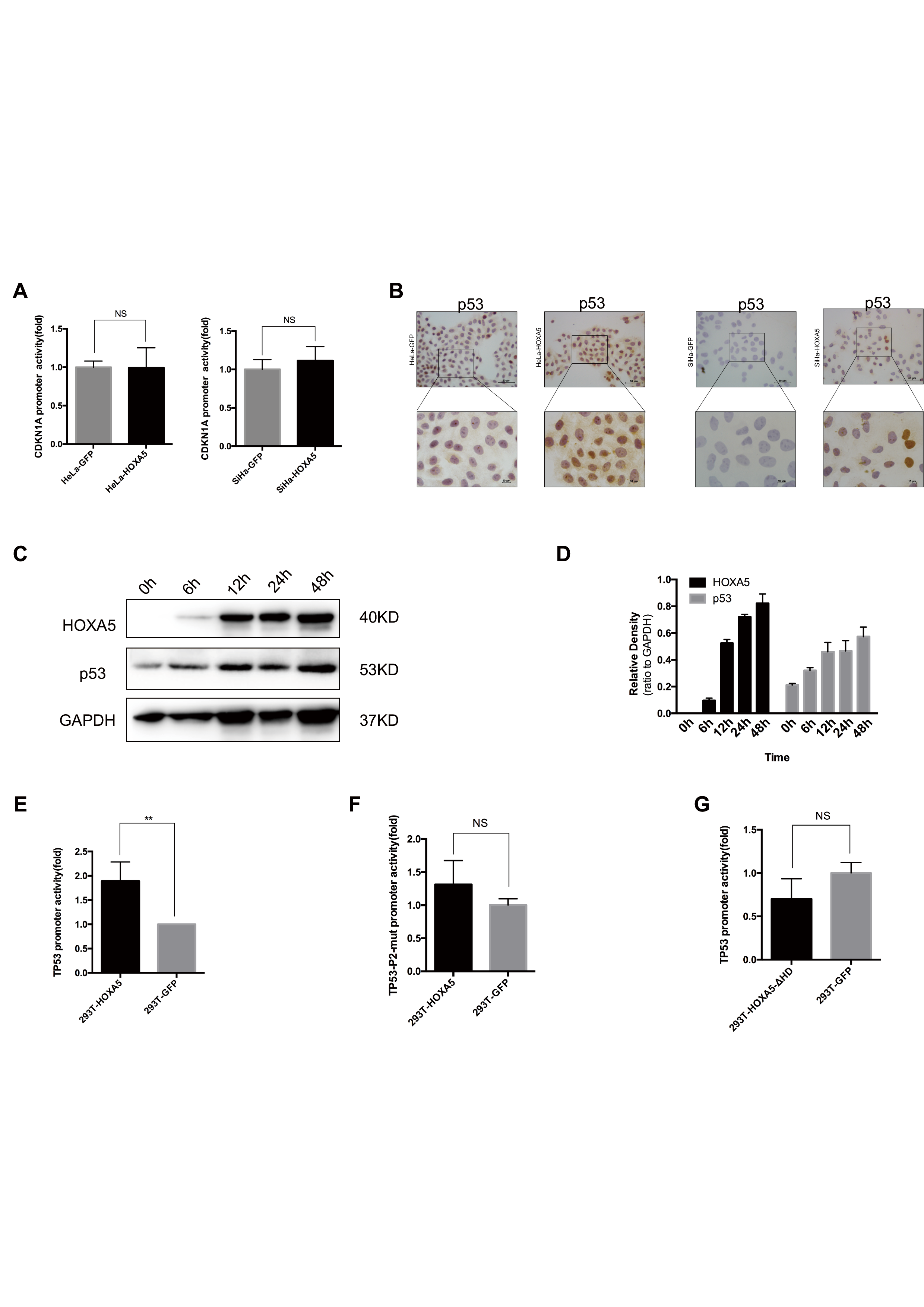

Supplement: Supplementary file 5 — Figure S5 [file 41419_2020_2629_MOESM5_ESM.png]

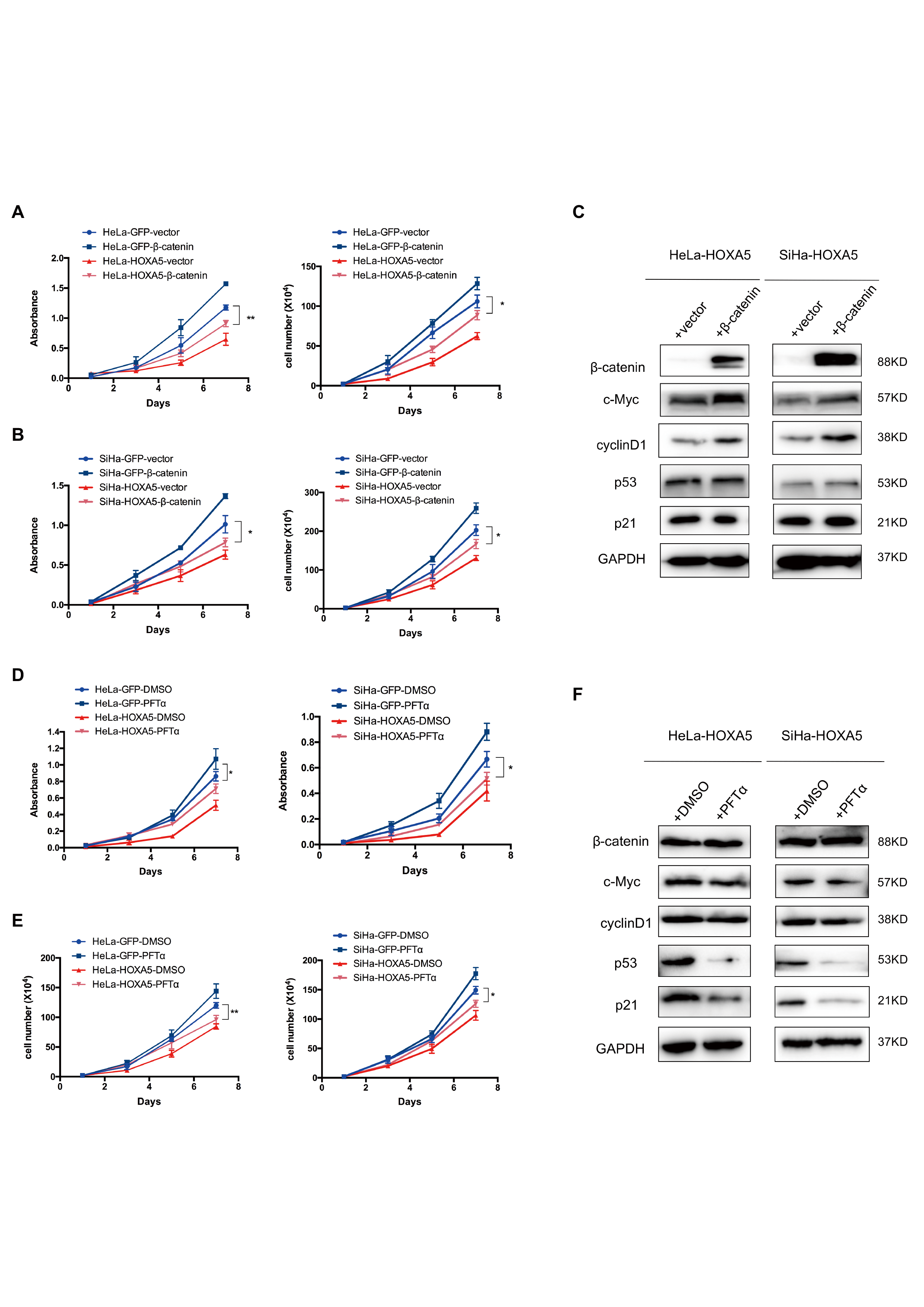

Supplement: Supplementary file 6 — Figure S6 [file 41419_2020_2629_MOESM6_ESM.png]
