# Supplementary material for: HOXA5 inhibits the proliferation and neoplasia of cervical cancer cells via downregulating the activity of the Wnt/β-catenin pathway and transactivating TP53
Source: Cell Death Dis. 2020 Jun 4;11(6):420. doi: 10.1038/s41419-020-2629-3 (PMC7272418; doi:10.1038/s41419-020-2629-3)
Supplement: Supplementary file 7 — Table S1 [file 41419_2020_2629_MOESM7_ESM.doc]

**Supplementary Table S1: HOXA5 expression levels in different tissue specimen**

| **Specimens** | **Total** | **HOXA5 Staining** | | *P* |
| --- | --- | --- | --- | --- |
|  |  | **Negative, No. (%)** | **Positive, No. (%)** |  |
| Normal | 42 | 10（23.8） | 32（76.2） |  |
| HSIL | 28 | 20（71.4） | 8（28.6） | < 0.001a |
| Carcinoma | 55 | 44（80.0） | 11（20.0） | NSb ,< 0.001c |

Abbreviation: HOXA5

Pearson 2-tailed chi-square test was used to determine the statistical significance of the level of expression of HOXA5 in different tissue specimens.

aNormal cervix versus high-grade squamous intraepithelial lesion.

bHigh-grade squamous intraepithelial lesion versus carcinoma.

cNormal cervix versus carcinoma.
