# Supplementary material for: HOXA5 inhibits the proliferation and neoplasia of cervical cancer cells via downregulating the activity of the Wnt/β-catenin pathway and transactivating TP53
Source: Cell Death Dis. 2020 Jun 4;11(6):420. doi: 10.1038/s41419-020-2629-3 (PMC7272418; doi:10.1038/s41419-020-2629-3)
Supplement: Supplementary file 8 — Table S2 [file 41419_2020_2629_MOESM8_ESM.doc]

**Supplementary Table S2: The list of primer sequences that used in this study**

| **Recombinant Vector Construction** | |
| --- | --- |
| HOXA5-CDS | F: CCGGAATTCGCCACCATGAGCTCTTATTTTGTAAACTCAT |
|  | R: CGCGGATCCAGATACTCAGGGACGGAAGGCCCCT |
| CTNNB-CDS | F: ATGGCTACTCAAGCTGATTTGATG |
|  | R: TTACAGGTCAGTATCAAACCAGGC |
| shHOXA5-112 | F:CACCGGACTACCAGTTGCATAATTATTCAAGAGATAATTATGCAACTGGTAGTCCTTTTTTG |
|  | R:GATCCAAAAAAGGACTACCAGTTGCATAATTATCTCTTGAATAATTATGCAACTGGTAGTCC |
| shHOXA5-1055 | F:CACCGCTTTCTGTTCATCTCTTTGTTTCAAGAGAACAAAGAGATGAACAGAAAGCTTTTTTG |
|  | R:GATCCAAAAAAGCTTTCTGTTCATCTCTTTGTTCTCTTGAAACAAAGAGATGAACAGAAAGC |
| shCtrl | F:CACCGTTCTCCGAACGTGTCACGTTTCAAGAGAACGTGACACGTTCGGAGAATTTTTTG |
|  | R:GATCCAAAAAATTCTCCGAACGTGTCACGTTCTCTTGAAACGTGACACGTTCGGAGAAC |
| **CRISPR** | |
| sgRNA1 | T: CACCGTCCCTGAATTGCTCGCTCA |
|  | B: AAACTGAGCGAGCAATTCAGGGAC |
| sgRNA2 | T:CACCAGGTACGGCTACGGCTACAA |
|  | B:AAACTTGTAGCCGTAGCCGTACCT |
| **RT-PCR** | |
| HOXA5 | F: AACTCATTTTGCGGTCGCTAT |
|  | R: TCCCTGAATTGCTCGCTCAC |
| GSK3B | F: GGCAGCATGAAAGTTAGCAGA |
|  | R: GGCGACCAGTTCTCCTGAATC |
| CTNNB | F: TCTGAGGACAAGCCACAAGATTACA |
|  | R: TGGGCACCAATATCAAGTCCAA |
| CCND1 | F: AAACAGATCATCCGCAAACAC |
|  | R: GTTGGGGCTCCTCAGGTTC |
| MYC | F: CCTGGTGCTCCATGAGGAGA |
|  | R: TCCAGCAGAAGGTGATCCAGAC |
| TP53 | F: CAGCACATGACGGAGGTTGT |
|  | R: TCATCCAAATACTCCACACGC |
| CDKN1A | F: TGTCCGTCAGAACCCATGC |
|  | R: AAAGTCGAAGTTCCATCGCTC |
| GAPDH | F: GCACCGTCAAGGCTGAGAAC |
|  | R: TGGTGAAGACGCCAGTGGA |
| **Luciferase Assays** | |
| TP53-P1  (-1835/+997bp) | F: GGAAGATCTTCCACAGTGAAAATCTCGG |
|  | R: CGACGCGTCGGCGGAGAATAG |
| TP53-P2 (-1071bp/+997bp) | F: GGAAGATCTTCCATGGCTTCGAAGTTC |
|  | R: CGACGCGTCGGCGGAGAATAG |
| TP53-P3  (-557bp/+997bp) | F: GGAAGATCTTCCGCTCTTACTTGCTACC |
|  | R: CGACGCGTCGGCGGAGAATAG |
| TP53-P4  (-380bp/+997bp) | F: GGAAGATCTTCCTAAAGTATCTGGGAGAAAAC |
|  | R: CGACGCGTCGGCGGAGAATAG |
| TP53-P5  (-277bp/+997bp) | F: GGAAGATCTTCCCAAAAGCTTTCTTCCT |
|  | R: CGACGCGTCGGCGGAGAATAG |
| TP53-P6  (-55bp/+997bp) | F: GGAAGATCTTCCCATGGCGACTGTC |
|  | R: CGACGCGTCGGCGGAGAATAG |
| TP53-P7  (+171bp/+997bp) | F: GGAAGATCTTCCCTGACTGAACTTGATGAG |
|  | R: CGACGCGTCGGCGGAGAATAG |
| TP53-P8  (+518bp/+997bp) | F: GGAAGATCTTCCCTGAGACTTTTGGATCTC |
|  | R: CGACGCGTCGGCGGAGAATAG |
| Cyclin D1 promoter region (-2024 bp-+200 bp) | F: CTAGCTAGCCGGGAAATCAACGAAGTTCCTAGTC |
|  | R: CCCAAGCTTAGAAACACCACGGCAAACTTCAAAG |
| CDKN1A promoter region (-1997 bp-+500 bp) | F: CCCAAGCTTAGTGTGGCCAAAGGATC |
|  | R: CGGGGTACCGTAATGAATTATTTAAATTTTTGTG |
| **Chromatin Immunoprecipitation Assay (ChIP)** | |
| S1 (-1071bp/-712bp) | F: ATGGCTTCGAAGTTCTCAGGGAT |
|  | R: GGCGCTTCTCGCCAAGATAGA |
| S2 (-711bp/-581 bp) | F: TACGCTCCCCCTACCGA |
|  | R: GAGGGAGACAGGTCTGAAGC |
| **Mutation** | |
| TP53-P2-mut | F: CGAGTCCCGCGGTAAGTCTTAAAGCACCTGCACC |
|  | R: GGTGCAGGTGCTTTAAGACTTACCGCGGGACTCG |
| HOXA5-ΔHD | F: CCGGAAGGCAAAAGGGCCGATAATAAGCTGAAAAGC |
|  | R: GCTTTTCAGCTTATTATCGGCCCTTTTGCCTTCCGG |
