# Supplementary material for: HOXA5 inhibits the proliferation and neoplasia of cervical cancer cells via downregulating the activity of the Wnt/β-catenin pathway and transactivating TP53
Source: Cell Death Dis. 2020 Jun 4;11(6):420. doi: 10.1038/s41419-020-2629-3 (PMC7272418; doi:10.1038/s41419-020-2629-3)
Supplement: Supplementary file 9 — Supplement Figure Legends [file 41419_2020_2629_MOESM9_ESM.docx]

**Supplement Figure Legends**

**FigureS1.Strategy for generation of human HOXA5 knockout clones based on CRISPR/Cas9-system and the generation of HOXA5 knockout clones in C-33A cell line.**

(A). Strategy for generation of human HOXA5 knockout clones based on CRISPR/Cas9 mediated editing system. B. Detailed insertion/deletion (indel) analysis of HOXA5-decient C-33A by DNA-sequencing of genomic PCR products. Consistent with the Western blot analysis, the analysis established the deletion of 56 nucleotides in cell clones.

**FigureS2. Overexpression of HOXA5 inhibited the growth of cervical cancer cells in vivo.**

(A)The total animals of xenograft experiment, HeLa-GFP cells on the left side and HeLa-HOXA5 cells on the right side. (B)Tumors obtained from (A). (C)The total animals of xenograft experiment, SiHa-GFP cells on the left side and SiHa-HOXA5 cells on the right side. (D)Tumors obtained from (C).

**FigureS3. HOXA5 affected the cell cycle transition through cyclinD1 and p21.**

The immunocytochemistry of cyclin D1(A) and p21(B) in HOXA5-modified HeLa and SiHa cells.

**FigureS4. HOXA5 repress the Wnt/β-catenin pathway**

(A) A luciferase construct containing the promoter sequence (−2040 bp~+200bp) of CCND1 was co-transfected with HOXA5 or GFP into 293T cells and the luciferase activity relative to Renilla control was measured. (B) HOXA5 repressed the transcriptional activation of the Wnt-dependent TCF reporter TOP Flash by either LiCl or β-catenin in 293T cells in a dose-dependent manner. (C- F) The quantitative analysis of the western blot in Fig.6K.(G) The expression of β-catenin and c-Myc in xenografts derived form HOXA5-modified HeLa cells. (H)The representative images of β-catenin and c-Myc in xenografts derived form HOXA5-modified HeLa and SiHa cells.

**FigureS5. HOXA5 up-regulated the expression of p21 by transacting TP53**

(A) A luciferase construct containing the promoter sequence (−1997 bp~+500bp) of CDKN1A was co-transfected into HOXA5-modified HeLa and SiHa cells, and the luciferase activity relative to Renilla control was measured. (B) The immunocytochemistry of p53 in HOXA5-modified HeLa and SiHa cells. (C) The expression of HOXA5, p53 and GAPDH in parental HeLa cells transiently transfected different time of HOXA5 plasmid by western blotting, and the quantitative analysis was shown in (D). (E)A luciferase construct containing the full-length promoter sequence (−1835 bp~+997bp) of TP53 was co-transfected with HOXA5 or GFP into 293T cells, and the luciferase activity relative to Renilla control was measured. (F) The pGL3.0-TP53- P2-mut plasmid was co-transfected with HOXA5 or GFP into 293T cells, and the luciferase activity was measured. (G)The pGL3.0-TP53- P2 plasmid was co-transfected with HOXA5-ΔHD or GFP plasmid into 293T cells, and the luciferase activity was measured.

**FigureS6. Up-regulating β-catenin or inhibiting p53 only partly rescue the proliferation-inhibition effect of HOXA5**

(A and B) The proliferation and viability of β-catenin transiently transfected HeLa-HOXA5 and SiHa-HOXA5 cells were detected by growth curves and MTT assay. (C)The expressions of β-catenin, c-Myc, cyclinD1, p53 and p21 were detected by western blot in β-catenin transiently transfected HeLa-HOXA5 and SiHa-HOXA5 cells. (D and E) The proliferation and viability of pifithrin-α (PFTα) -treated HeLa-HOXA5 and SiHa-HOXA5 cells were detected by growth curves and MTT assay. Pifithrin-α (PFTα): the specific TP53 inhibitor. (F)The expressions of β-catenin, c-Myc, cyclinD1, p53 and p21 were detected by western blot in pifithrin-α (PFTα)-treated HeLa-HOXA5 and SiHa-HOXA5 cells.
